# A hybrid approach using entropy and TOPSIS to select key drivers for a successful and sustainable lean construction implementation

**DOI:** 10.1371/journal.pone.0228746

**Published:** 2020-02-05

**Authors:** Gholamreza Dehdasht, M. Salim Ferwati, Rosli Mohamad Zin, Nazirah Zainul Abidin

**Affiliations:** 1 School of Civil Engineering, Faculty of Engineering, Universiti Teknologi Malaysia, Skudai, Malaysia; 2 Department of Architecture and Urban Planning, College of Engineering, Qatar University, Doha, Qatar; 3 School of Housing, Building and Planning, Universiti Sains Malaysia, Penang, Malaysia; Institute for Advanced Sustainability Studies, GERMANY

## Abstract

Successful implementation of the lean concept as a sustainable approach in the construction industry requires the identification of critical drivers in lean construction. Despite this significance, the number of in-depth studies toward understanding the considerable drivers of lean construction implementation is quite limited. There is also a shortage of methodologies for identifying key drivers. To address these challenges, this paper presents a list of all essential drivers within three aspects of sustainability (social, economic, and environmental) and proposes a novel methodology to rank the drivers and identify the key drivers for successful and sustainable lean construction implementation. In this regard, the entropy weighted Technique for Order of Preference by Similarity to Ideal Solution (TOPSIS) was employed in this research. Subsequently, an empirical study was conducted within the Malaysian construction industry to demonstrate the proposed method. Moreover, sensitivity analysis and comparison with the existing method were engaged to validate the stability and accuracy of the achieved results. The significant results obtained in this study are as follows: presenting, verifying and ranking of 63 important drivers; identifying 22 key drivers; proposing an MCDM model of key drivers. The outcomes show that the proposed method in this study is an effective and accurate tool that could help managers make better decisions.

## Introduction

The construction industry is one of the largest and most important sectors in many countries [1]. Unfortunately, many industrial countries, such as Malaysia, face problems during the construction lifecycle. These problems have an enormous effect on human health, the natural environment, and the economy [2–4]. There are opportunities, however, to promote sustainability in construction. To overcome these problems, parties involved in construction projects have exerted great effort to implement strategies that could lead to sustainability and a greener environment for a better future of the world [5, 6].

Lean construction is a practical approach to enhancing construction sustainability. It includes clearly defined goals for the process of delivery [2] that differentiates it from other construction management methods. It tends to deliver process, production control, and concurrent design and process during a project’s lifecycle [7]. Toyota developed the term "lean" and influenced by Total Quality Management (TQM) to reduce machine setup time. In this regard, a production system was designed with a set of objectives, which include: (1) waste elimination–deleting anything that does not add value, (2) establishment of a production system with a continued flow, (3) consistent flow and perfect product through decision making and information sharing, and (4) flow perfection–meeting customers’ requirement on time with zero inventory [8]. Koskela [9], reports on the adoption of lean production concepts in the construction industry in which production was idealized into three corresponding ways, including transformation, flow theory of production, and value generation (TFV). This multilateral perception of production gave birth to lean construction as a discipline [5].

Recently, several studies have been done to demonstrate the interrelationship between lean construction and sustainability [2, 10, 11]. According to Nahmens and Ikuma [2], lean construction is an approach to promote sustainability by optimizing the utilization of resources and human safety procedures during construction and reducing waste through the implementation of standard procedures. Therefore, the aims of lean construction are waste reduction, continuous improvement, high levels of user focus, improved commitment and communication, value for money, and the betterment of both project management and supply chain [12]. Womack and Jones [13], suggest that lean production is particularly capable of reducing cycle time as well as decrease the total and indirect costs, while at the same time, retain the quality standards. Black [14], states that lean construction stems from the goal of a lean production system, which is maximizing value, minimizing waste, and specifying methods and their application in new projects. Mitropoulos et al. [15] mention how the lean approach could have positive effects on safety. Nahmens and Ikuma [16] test this by proposing a new model to show the influence of lean construction on safety programs and initiating a new model using the Continue Improvement (CI) program. The program asks how a continuous improvement program can trim down the opportunities for accidents through eliminating waste (in materials, motions, and process steps), and therefore, reducing safety hazards. Fu et al. [17], investigated the link between lean construction and the environment. They presented how lean construction can help decrease energy usage and air pollution by improving efficiency and productivity during construction projects and reducing construction waste. This study, therefore, focuses on lean construction as a sustainable approach in the construction stage.

Lean construction has been applied with substantial income in various countries such as South Africa [18], Chile [19], Turkey [20], Brazil [21], Singapore [22], the Netherlands [23], and USA [2]. It is understandable to achieve lean construction in construction projects as the need to adapt this concept can cover all aspects of sustainability (social, economy, and environment), although the implementation and adaptation process are impartially poor with either low rates or no progress at all [24, 25]. Several countries have had problems with the lean process during construction projects [26–29] because the lean culture within these countries is lower than the expectation [27]. Developed countries have a well-defined roadmap and strict policies that guarantee efficient and effective implementation of strategies such as lean management and sustainability [30]. While in developing countries such as Malaysia, the research frameworks of such strategies are rare, and the implementation is still in the initial stage [10]. Beyond this, cultures, environmental conditions and other circumstances, barriers and drivers will vary from country to country [31]. These apparent shortages exist in many studies of the successful implementation of the lean process. Therefore, to achieve successful lean construction implementation, it is necessary that the industrial practitioners consider a feasible country-specific approach to overcome these barriers. According to Ogunbiyi [11], to tackle these barriers, it is highly significant to ascertain the factors that strongly motivate or force management to implement lean construction successfully. The motivating factors are called drivers and they can motivate organizations, companies, managers, and decision-makers to implement a lean strategy.

Based on the Business Dictionary, a “driver” signifies the condition, resource, process, or decision that is vital for the continued success and growth of a business. Understanding key drivers can hence effectively help to implement lean construction successfully by motivating managers, employees, stakeholders, and persons involved in construction projects. Despite the key role of drivers for successful implementation and adapting lean construction within the construction industry, there are not enough research studies on identifying the essential drivers for successful and sustainable lean construction implementation. Additionally, in the context of lean construction, there is an insufficient attempt to primarily focus on identifying a methodology to rank the important drivers and understand the key drivers. According to Leong and Tilley [32], the lack of identification of factors affecting the successful implementation of lean construction has led to the inability of organizations to recognize efforts that should be improved, where these efforts should be focused, or which effort is needed to obtain the best result. This research is, therefore, an effort to fill these gaps by ranking drivers and identifying key drivers that have critical roles in successful lean construction implementation with a sustainability approach. For this reason, this study employs the Multi-Criteria Decision Making (MCDM) method to meet its objectives.

The objective of this study is to identify and classify all the crucial drivers of lean construction implementation in three aspects of sustainability (social, economic, and environmental). Furthermore, this study aims to propose a new methodology that helps to identify the critical drivers for sustainable lean construction implementation. In this regard, this paper proposes a Multi-Criteria Decision Making (MCDM) model of essential drivers, which can help to adapt and implement lean construction successfully with a sustainability approach in Malaysia. This study employs the Technique for Order of Preference by Similarity to Ideal Solution (TOPSIS) method to rank all the crucial drivers as well as to identify the critical drivers of lean construction implementation based on their effect in approaching each aspect of sustainability. The entropy method is used to calculate the weight of all the criteria in TOPSIS computation, which can effectively avoid the effects of human subjective factors.

TOPSIS is a well-known technique to deal with the ranking problem of alternatives from the best to worst. The main porous of TOPSIS is that the preferred option should be the closest to the positive ideal solution and the furthest from the perfect negative solution. The ideal resolution is, therefore, the solution that not only maximizes the benefit criteria but also minimizes the cost criteria. In other words, the ideal solution contains all the highest values of the available criteria, while the negative ideal solution has the worst values of the possible criteria [33]. One of the main advantages of the TOPSIS approach is that it delivers influence results for the ranking of alternatives that have absolute data for each indicator [34]. Dos Santos et al. [35] suggest that the integration of TOPSIS with other MCDM approaches may solve problems more efficiently and flexibly. On the other hand, Shannon’s Entropy is recommended to calculate the weight of the criteria since it is an effective method that makes decision-making more reliable and accurate with no significant modeling difficulties [36]. According to Li et al. [37], evaluation of the weights of indexes through subjective weight methods such as the survey method, Delphi method, Analytic Hierarchy Method (AHP), etc., could lead to deviations of indexes’ weights due to subjective factors.

On the contrary, objective fixed weight methods such as entropy could effectively eliminate human-made disturbances because they are conducted according to the inherent information of indexes and define the weight of indexes, which make results consistent with facts (Jozi et al., 2012). The integration of entropy and TOPSIS can therefore effectively help increase the reliability and accuracy of driver ranking. To illustrate the performance and efficiency of this hybrid method, an empirical study was conducted within the Malaysian construction industry. Sensitivity analysis and comparison with an existing tool in MCDM methods for ranking the alternatives are used to validate the stability and accuracy of the final results.

In summary, there is a need to identify drivers that help successful lean construction implementation within the construction industry. It is then necessary to introduce a methodology for ranking drivers and subsequently to identify key drivers. The proposed method in this study not can only be used by other studies in identifying key barriers or drivers based on their country, a specific project or other circumstances, but can also be applied in other fields of science for ranking factors to successful and sustainable implementation strategies. The identified key drivers from different dimensions can help managers, decision-makers, and policymakers to concentrate on essential drivers. These key drivers ultimately give them the insight to select the best strategy for successful lean construction implementation with a sustainability approach. The proposed MCDM model of key drivers in this study can be a reference for comparison with the identified key drivers by future studies in developing nations. In this regard, this study aims to address the following questions:

1. What are the essential drivers for successful and sustainable lean construction implementation?
2. What would be the result of applying entropy weighted TOPSIS in identifying key drivers to a successful and sustainable lean construction implementation?

The rest of this paper is structured like the following. Section 2 presents the drivers of lean construction implementation, including the most important drivers and classification groups, as well as entropy and TOPSIS methods. Section 3 offers a hierarchical structure of the most important drivers for successful and sustainable lean construction implementation. This section also includes the entropy weighted TOPSIS calculation outcomes as well as the results of this research, including the key drivers’ hierarchy model for implementing successful and sustainable lean construction. Finally, Section 4 is a discussion, and section 5 is the conclusion.

## Drivers of lean construction implementation

Various studies indicate that understanding the drivers of lean implementation helps to adapt and execute a successful process. This section, therefore, attempts to identify critical drivers through an extensive literature review. It first evaluates, however, the concept and aim of identifying the key drivers presented below.

Chou and Pramudawardhani [38] define key drivers as the motivation index that indicates the chance of success for any project. Identifying key drivers has a fundamental role in successful implementation and adapting methods (i.e., lean construction, risk management, value engineering, etc.) [39]. A general view of drivers is necessary to understand how methods (i.e., lean construction, risk management, value engineering, etc.) can be made more successful and popular [40]. Renault et al. [41] argue that understanding drivers would help management acquire the necessary support for the successful implementation of a program or method. According to Ilić and Nikolić [42], better awareness of reliable drivers helps to develop and improve a method of execution. In the context of lean, Singh Sangwan et al. [43] define the drivers of lean implementation as factors that contribute to the easy adoption of the lean method in the industry. Hence, understanding key drivers are essential for the adaptation and successful implementation of any method (i.e., lean construction, risk management, value engineering, etc.). This study attempts to identify the key drivers in lean construction that can potentially help future studies in the successful implementation of lean construction.

**Fig 1:**
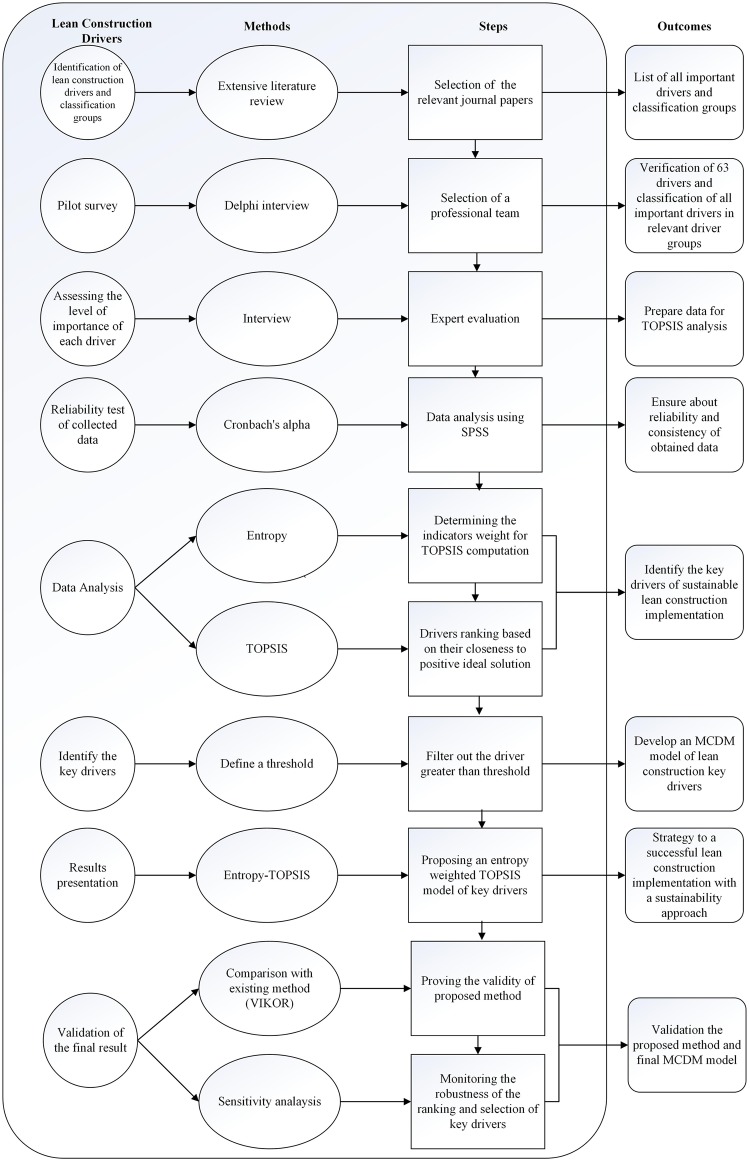
Research flow for MCDM model of key drivers.

This section has two subjects. The first presents the most important drivers and classification groups of lean construction as well as discussing all related works in the field of drivers of lean construction implementation. The second introduces the concept of entropy and Technique for Order of Preference by Similarity to Ideal Solution (TOPSIS). An overview of the process of the proposed model using entropy weighted TOPSIS is shown in Fig 1. As shown in Fig 1, the research flow chart consists of six main steps, which include a literature review to identify the significant drivers and classification groups, pilot survey for the verification and classification of all-important drivers, data collection to assess the level of importance of each driver, TOPSIS analysis to rank all-important drivers, identifying the key drivers through defining a threshold, and finally, a proposal of a model of key drivers for successful sustainable lean construction implementation.

### Identification of lean construction drivers

Identifying the drivers of lean construction implementation was the first objective of this study. The goal is to prepare a list of capable drivers that can motivate organizations into the successful implementation of the lean concept during its projects. Despite various studies within the context of lean construction, few attempted to identify the drivers for implementing lean construction. Those studies found only a few and without any statistical analysis to understand the key drivers. To overcome these limitations, this study considers the following strategies; (1) identification of drivers from previous studies (that investigated drivers of lean construction implementation), (2) a review on the academic publications in the field of lean construction and the extraction of influent drivers that might not be directly pointed out as a driver, (3) making use of the significant drivers of lean manufacturing implementation as is mentioned by academic publications, and (4) an investigation of classification groups for barriers of lean construction implementation to find the best classifications. The main reason for reviewing the classifications of both drivers and barriers is due to the proximity of these factors. The subsequent section explains all capable drivers and classification groups.

Several studies have sought to explain the benefits of lean construction implementation. Section 1 investigates the linkage between lean construction and sustainability and outlines the benefits of implementing the lean process within the three dimensions of social, economic, and environmental. These benefits can be drivers for managers, stockholders, and governments to apply the lean process for their projects or industries. Some studies focus on the effect of lean principles and techniques as well as the gained benefits [8, 44, 45], while others concentrate on the successful implementation of lean construction and its positive effect on the three dimensions of sustainability [2, 10, 46]. Most of these studies give extra attention to the impact of lean construction in economic aspects such as improved productivity and efficiency as well as decreased cost and project duration, etc.

On the contrary, other studies were done recently to demonstrate the benefit of implementing the lean process on both social and environmental dimensions. Salem et al. [44], present how lean techniques can improve transparency, process variability, flow variability, and continuous improvement. Ikuma et al. [47] show that lean construction not only improves productivity but also reduces or eliminates safety and ergonomic hazards in practical design and improves process layout. Furthermore, Issa [48] evaluates the effect of implementing lean construction on project risks. The results prove that lean construction minimizes and mitigates the impact of a majority of risk factors in projects. Kim and Bae [49] present how lean construction can help to decrease using energy and air emissions, such as CO, CO2, NO2, PM10, SO2, and HC, by improving the supply chain as well as using JIT and prefabrication tools.

Subsequently, some studies have proposed and classified the drivers of lean process implementation. Most of these studies have extracted the said drivers from the benefit of implementing the lean process, which was explained in previous studies. Ogunbiyi [11], in her Ph.D. thesis about the implementation of lean in sustainable construction projects within the UK, introduces 31 drivers and divides according to the three dimensions, the economic, social, and environmental. Ametepey et al. [50] introduce 17 drivers of implementing lean construction in the South African construction industry and rank them through mean index. The study excludes the classification of drivers and presents a methodology for identifying key drivers. The results of the said study show that continuous improvement promotes communication and improves quality, and thus, is the most important driver of lean in South Africa. Gandhi et al. [30] introduce 15 drivers of integrated lean-green manufacturing for Indian manufacturing SMEs and categorize them into internal, economy and market, policy, and society drivers, where each group is a subset of one dimension of sustainability. The outcome reveals that current legislation, upgrading of technology, and top management commitment are the most important drivers for successful lean-green manufacturing implementation in India. Singh Sangwan et al. [43], identify the drivers of lean management implementation in Indian ceramic industries. They introduce 20 key drivers and divide them into three categories; internal, external, and policy drivers.

On the other hand, Sohal and Egglestone [51] investigate the drivers of lean production within the manufacturing sector of Australia and presented 24 drivers. Zhou [52], proposes 13 key drivers of lean practices in Small- and Medium-sized Enterprises (SMEs) in the US to adapt and have a successful implementation of lean in SMEs. Nordin et al. [53], evaluate the barriers and drivers of lean implementation within the manufacturing sector of Malaysia. They propose five main groups of drivers and barriers; process and equipment, planning and control, human resources, supplier relationships, and customer relationships.

Contrary to the drivers of lean construction, there are several studies in the field of barriers to lean construction implementation. These studies classify restrictions in several classification groups. Sarhan and Fox [27], divide barriers of lean construction implementation in the UK into nine significant groups; fragmentation and subcontracting, procurement and contracts, culture and human attitudinal issues, adherence to traditional management concepts, financial matters, management commitment, and support, lean awareness/ understanding, educational issues, and customer-focused. Bashir et al. [26], in their review on barriers preventing the sustainable implementation of lean construction in the UK, present six classifications of barriers; management, financial, education, government, technical, and human. Alinaitwe [54], in research about lean construction barriers in Uganda’s construction industry, presents 40 barriers and classifies them into ten groups: teamwork, total quality management (TQM), benchmarking, variability, simplification, flow reliability, JIT, pull scheduling, concurrent engineering, and Business Process Re-engineering (BPR). Marhani, et al. [55] present seven main barrier groups in lean construction implementation within Malaysia, which includes management, technical, financial, human attitude, the process of lean construction, education, and government. Finally, Asri et al. [56] propose six specific barrier groups of lean construction implementation in Malaysia; management, financial, educational, governmental, technical, and attitudinal aspect.

Table 1 presents all identified drivers. As was mentioned, this study attempts to identify all possible drivers of lean construction implementation, which covers the three aspects of sustainability. The study not only presents drivers with a general concept but also attempts to investigate all possible drivers in detail, which is critical for the successful implementation of lean construction. For this reason, it may seem that some drivers’ concept is linked together or are overlapping in theory. This study tries to explain every single driver to show the limitations between them (S1 File). This detailed information of drivers helps to investigate the root causes of problems and provide an overview to managers, stockholders, and any persons who are involved in the construction industry and how they should respond and act accordingly. To this extent, this study collects and reviews all significant drivers from academic publications related to drivers of the lean concept in industry and construction. Table 1 presents a total number of 63 identified drivers of lean construction that collected from previous studies.

**Table 1:** List of identified drivers.

Code	Lean construction drivers	References
**c1**	Improve scheduling	[8, 51, 57]
**c2**	Improve planning	[58–60]
**c3**	Global competition	[61–63]
**c4**	More focused on organization structure	[8, 44, 51]
**c5**	Promote prompt and reliable delivery to the customer	[48, 51, 52, 64, 65]
**c6**	Short time to fulfill customer orders	[53, 66–68]
**c7**	Promote ability in frequent changes in order by customers	[43, 64, 69]
**c8**	Meeting customers’ expectation	[8, 12, 69, 70]
**c9**	Improve process control	[8, 51, 71–73]
**c10**	Improve the production capacity of the company	[51, 52, 67]
**c11**	Reduce management levels	[44, 57, 69]
**c12**	Increase market share	[12, 53, 62, 74–76]
**c13**	Increase flexibility	[12, 51, 52]
**c14**	Reduce high-labor-cost or labor requirements	[8, 51, 64]
**c15**	Cost savings (to finish objectives lower than historical cost)	[2, 43, 44, 46]
**c16**	Cost reductions (to remove unwarranted expenses)	[8, 44, 57, 62]
**c17**	Efficiency improvement	[51, 60, 77]
**c18**	Optimization	[5, 12, 63]
**c19**	Improve profit margin	[45, 52, 78, 79]
**c20**	Mitigation of project risk	[2, 47, 48, 80]
**c21**	Competitive advantage	[62, 81–84]
**c22**	Improve-manpower productivity	[43, 66, 85, 86]
**c23**	Multiskilling of the workforce	[44, 51, 87, 88]
**c24**	Improve capabilities (department, organization, person, system)	[43, 51, 64, 67, 71]
**c25**	Promote skilled workers	[51, 64, 66]
**c26**	Commitment to self-action teams	[8, 51, 86, 89]
**c27**	Continuous improvement	[2, 12, 90]
**c28**	Improve safety	[12, 16, 60, 91, 92]
**c29**	Enhanced organization’s reputation	[11, 93–96]
**c30**	Facility of understanding the concepts of lean construction	[7, 8, 87, 96, 97]
**c31**	Promote awareness of some or all tools and techniques	[11, 44, 96]
**c32**	Employee autonomy	[7, 11, 98, 99]
**c33**	Improve low-quality materials/parts by suppliers	[54, 64, 67, 100, 101]
**c34**	Improve on-time delivery by the supplier	[54, 64, 67, 100, 102]
**c35**	Improve supply reliability	[54, 81, 100, 101, 103]
**c36**	Reduction in inventory	[51, 52, 64, 67, 104]
**c37**	Reducing spare parts inventory	[10, 11, 51, 60]
**c38**	Improve coordination between supplier and company	[54, 81, 100, 101]
**c39**	Reduce lead times	[44, 51, 105, 106]
**c40**	Redesign of processes	[16, 51, 69, 71]
**c41**	Improve the commitment of employees	[51, 53, 66, 89, 107, 108]
**c42**	High-product variety	[44, 51, 52, 66, 68, 109]
**c43**	Improve workplace organization	[8, 43, 67, 71]
**c44**	Improve standard operating procedures	[11, 51, 52, 65]
**c45**	Reduce steps of project’s life cycle	[8, 69, 82, 110]
**c46**	A stronger focus on performance	[8, 12, 47, 82]
**c47**	Improved process layouts	[16, 44, 51, 71]
**c48**	Improve self-criticism	[8, 19, 60, 110, 111]
**c49**	Improve transparency among team	[2, 44, 82, 88, 112]
**c50**	Reduce leadership conflict	[26, 110, 113, 114]
**c51**	Improve teamwork	[2, 8, 60, 65, 82]
**c52**	Improve company culture	[5, 46, 65, 96, 115–117]
**c53**	Increase trust	[7, 46, 65, 81, 114, 115]
**c54**	Improve information sharing	[44, 118–120].
**c55**	Motivate employees and shape their behavior	[8, 65, 76, 113, 118, 121, 122].
**c56**	Improve housekeeping	[2, 55, 110, 123].
**c57**	Increase employee morale	[8, 12, 46, 65, 110, 124]
**c58**	Government policy and regulation	[10, 12, 125]
**c59**	Reduce air pollution	[10, 49, 126–129]
**c60**	Keep the environment through reduction of construction waste	[2, 11, 46, 60, 128]
**c61**	Reduction in material usage	[10, 11, 46, 47, 110, 128]
**c62**	Water efficiency	[11, 46, 128, 130]
**c63**	Reduction in energy consumption	[10, 11, 46, 128, 130]

### The TOPSIS method

The TOPSIS method, first developed by Hwang and Yoon [131], is one of the well-known Multi-Criteria Decision Making (MCDM) methods to identify a solution from a finite set of points. TOPSIS is a linear weighting technique and is the abbreviation for Technique for Order Preference by Similarity to Ideal Solution. TOPSIS can be used with both fuzzy and numbers. The method is based on defining both positive and negative ideal solutions and subsequently ranking their feasibility according to the furthest distance from the negative ideal solution and the shortest distance from the positive ideal solution. TOPSIS also presents an index called closeness to the positive-ideal solution and remoteness from the negative-ideal solution, in which the alternative with the highest similarity to the positive-ideal solution should be chosen [34, 132].

Today, many researchers in the field of construction have to use TOPSIS for ranking the factors. For example, Gandhi et al. [30] employ TOPSIS methods to rank the drivers for integrated lean-green manufacturing. Vinodh and Swarnakar [133], engage the TOPSIS method to select the optimal Lean Six Sigma (LSS) projects for an automotive component manufacturing organization. Kabirifar and Mojtahedi [134], use TOPSIS to rank Engineering, Procurement, and Construction (EPC) critical activities across large-scale residential construction projects in Iran. Zavadskas et al. [135] review 105 papers that propose, present, develop, and extend the TOPSIS method for solving decision-making problems from 2000 to 2015. Their study concludes that TOPSIS was developed or extended by 49 scholars and 56 scholars presented or proposed new modifications for issues related to TOPSIS methods. Liang et al. [136], propose a new method for three-way decisions using ideal TOPSIS solutions for Pythagorean fuzzy information. The study employs TOPSIS to estimate the conditional probability of Pythagorean Fuzzy Decision-Theoretic Rough Sets (PFDTRSs) by determining ideal solutions. Tian et al. [137], show the flexibility of MCDM techniques for developing, integrating, and modifying by proposing a novel Choquet integral-based grey comprehensive evaluation (GCE) method to assess MCDM problems with many qualitative and interactive indices.

The mentioned examples show the effectiveness and workability of the TOPSIS method for selecting critical indicators. Despite this importance, however, there is not adequate attention paid in the field of lean construction to using MCDM techniques such as the TOPSIS method for choosing the best solutions. The procedure of the TOPSIS concept involves the following steps [131]:

**Step 1:** The construction of a decision matrix.
  Given a set of alternative *V* = {*V*_*i*_ | *i* = 1, 2, …, *m*} and a set of criteria *C* = {*C*_*j*_ | *j* = 1, 2, …, *n*}, where *X* = {*x*_*ij*_ | *i* = 1, 2, …, *m*; *j* = 1, 2, …, *n*} represents the decision matrix and *x*_*ij*_ is the value of *i*th alternative with respect to *j*th indicator.
**Step 2:** The computation of aggregate ratings for the alternatives and the criteria
  To combine the opinions of all experts and achieve the matrix *Z* = [*a*_*ij*_] which is an average rating of other options based on the requirements, in the next step, the average of each respondent’s scores is computed using Eq 1:
  $$a_{ij}=\frac{1}{k}\sum_{k=1}^{k}x_{ij}^{k}$$ (1)
**Step 2:** The normalizing of the decision matrix.
  To avoid the effect of the index dimension and its variation ranged on assessment results, the normalization of the original matrix is required to certify that all the attributes are in the same format and equivalent. Therefore, normalized values can be calculated using Eq 2:
  $$r_{ij=\frac{a_{ij}}{\sqrt{\sum_{i=1}^{m}a_{ij}^{2}}}}(i=1,\ldots ,m;j=1,\ldots ,n)$$ (2)
**Step 3:** The determination of the weighted normalized decision matrix.
  In this section, a set of weights of *n* indicators W = {*w*_*j*_ | *j* = 1, 2, …, *n*}, where *w*_*j*_ > 0 and $\sum_{j=1}^{n}w_{j}=1$, is applied to compute the weighted normalized decision matrix by Eq 3:
  $$v_{ij}=w_{j\;}r_{ij}$$ (3)
**Step 4:** The determination of the ideal solution (positive and negative).
  The positive ideal solution consists of the optimum values of every attribute from the weighted normalized decision matrix, while the negative ideal solution has the worst value of every attribute from the weighted normalized decision matrix calculated as follows:
  $$A^{+}=\left\{v_{1}^{+},\ldots ,v_{j}^{+},\ldots ,v_{n}^{+}\right\}=\{({\text{max}}_{i}\;v_{ij\;}|j\epsilon J_{1}),({\text{min}}_{i}\;v_{ij\;}|j\epsilon J_{2})|1\ldots ,m\}$$ (4)
  $$A^{-}=\left\{v_{1}^{-},\ldots ,v_{j}^{-},\ldots ,v_{n}^{-}\right\}=\{({\text{min}}_{i}\;v_{ij\;}|j\epsilon J_{1}),({\text{max}}_{i}\;v_{ij\;}|j\epsilon J_{2})|1\ldots ,m\}$$ (5)
  Where, *J*_1_ is the set of benefit criteria and *J*_2_ is the set of cost criteria.
**Step 5:** The calculation of the separation value.
  The separation value subsequently is obtained by calculating the distance between each alternative as well as the positive and negative ideal solutions by respectively using Eqs (6) and (7):
  $$D_{i}^{+}=\sqrt{\sum_{j=1}^{n}{(v}_{ij}-v_{j}^{+}{)}^{2}}i=1\ldots ,m.$$ (6)
  $$D_{i}^{-}=\sqrt{\sum_{j=1}^{n}{(v}_{ij}-v_{j}^{-}{)}^{2}}i=1\ldots ,m.$$ (7)
**Step 6:** The determination of the closeness coefficients and ranking the alternatives.
  The closeness coefficients of each alternative are calculated by Eq (8):
  $${CC}_{i}=\frac{D_{i}^{-}}{D_{i}^{+}+D_{i}^{-}}$$ (8)
  Where 0 < *CC*_*i*_ ≤ 1, *i* = 1, …, *m*.

Finally, the alternatives can be ranked based on the closeness coefficients, in which the best alternative is the one with the highest value.

### Entropy weight vector calculation

The entropy weight method is an effective method to accurately weigh the relative importance of the identified criteria for TOPSIS computation [138]. This method first developed from thermodynamics to information systems [139]. The concept of ‘‘information entropy” includes the uncertainty of signals in communication processes [140]. The base of the entropy weight method is the volume of information to calculate the index’s weight, which is similar to the main objective of fixed weight methods [37].

In previous studies, the criteria weight determined by the evolution of experts in TOPSIS method. The results for calculating weight in this method are quite subjective, in which the subjective factors have a higher undesirable effect on the evaluation result [17]. For this reason, the entropy method should be employed to compute an actual weight within the weighting process of the evaluation indicator system, and therefore, the effect of human subjective factors can be avoided [141]. According to Wang et al. [138], this objective weighting process can overcome the shortage of subjective weighting method, as the method is based solely on neutral data. Therefore, this paper has employed the information entropy method to determine the criteria weight. The following summarizes the basics of Shannon entropy weighting process:

**Step 1:** The normalizing of the available decision matrix. Suppose that the decision matrix of A = (*x*_*ij*_)_*m*×*n*_ With m alternative and n criteria is available. The decision matrix is, therefore, normalized by Eq (9):
  $$P_{ij}=\frac{x_{ij}}{\sum_{i=1}^{m}x_{ij}}$$ (9)
**Step 2:** The calculation of entropy for each index.
  $$E_{j}=-\frac{1}{Lnm}\sum_{i=1}^{m}P_{ij}Ln\;P_{ij,}\;j=1,\ldots ,n$$ (10)
**Step 3:** The calculation of the degree of deviation of essential information for each criterion.
  $$D_{j}=1-E_{j\;}j=1,\ldots ,n$$ (11)
  Where *D*_*j*_ measures the degree of deviation of essential information for the *j*th criteria.
**Step 4:** The calculation of the criteria’s entropy weight.
  $$w_{j}=\frac{D_{j}}{\sum_{j=1}^{n}D_{j}}$$ (12)
  Where *w*_*j*_ is the importance weight of the *j*th criteria.

## Empirical study

An empirical study was conducted in this study to verify all identified drivers and classification groups, also to validate this integrated method, i.e., TOPSIS and Entropy. In the following sections, the hierarchical structure of lean construction drivers, data collection, and analysis process has been discussed respectively.

### Hierarchical structure of lean construction drivers

Through the extensive literature review in the previous section, a large number of 63 drivers and the essential classification groups identified. All identified drivers were evaluated and verified through a pilot survey. Before distributing the actual questionnaire, a pilot survey was conducted to assess the feasibility and sensibility of the survey. A semi-structured format of Delphi interview conducted with seven Malaysian construction professionals, who were aware of the lean process, in which all drivers accurately evaluated and verified (S2 File). The interviewees were mostly amongst senior experts with an average of 15 years of experience. Pilot interview conducted in three rounds. After the third round, the pilot interview discontinued because of the strong consensus. During the interview, all classification groups of drivers and barriers evaluated. In fact, through interviews and discussions with experts, seven classification groups (management, financial, technical, resource, awareness and education, environmental, people, and culture) were selected, and all drivers classified within the relevant groups. Besides, the relation between the elements as well as the sufficiency, degree of difficulty and level of clarity of the questions assessed.

**Fig 2:**
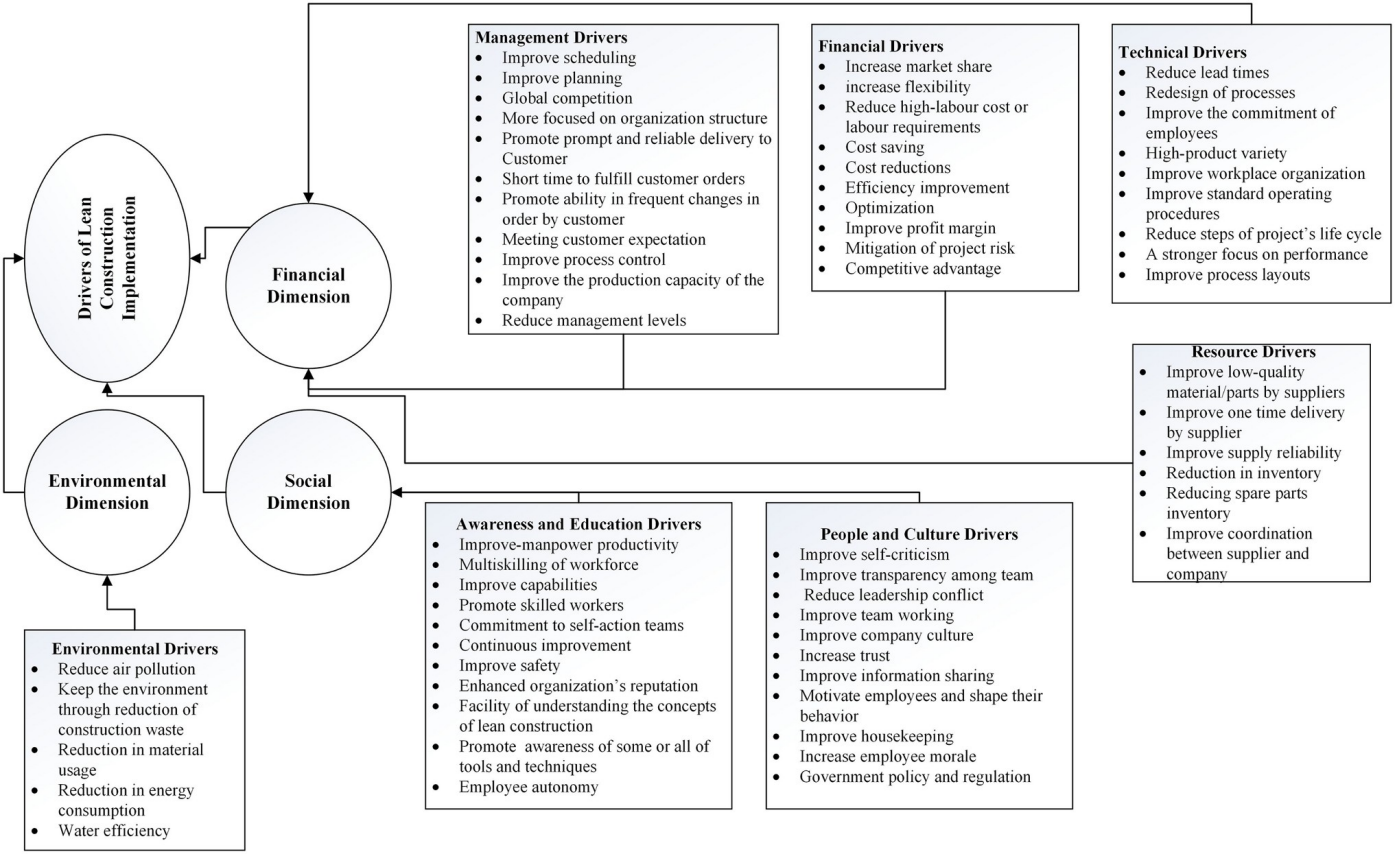
A hierarchical structure of drivers for a sustainable lean construction implementation.

Additionally, the classifications of drivers helped to improve the design of the questionnaire and also promoted the reliability and workability of the questionnaire. Also, understanding the classification groups facilitated the evaluation of the important relationship between its key drivers. Fig 2 shows The ultimate hierarchical structure of the important drivers for the implementation of lean construction projects, and it illustrates that the presented drivers could be classified under the three aspects of sustainability. In other words, these drivers are helpful to achieve sustainable lean construction implementation.

The following section will evaluate the proposed methodology for identifying key drivers. The outcome of this section helps the decision-making procedure by prioritizing the important drivers of lean construction implementation and also aid in the successful implementation and adaption of sustainable lean construction within construction projects.

### Data collection

In this study, entropy weighted TOPSIS was utilized to rank all the important drivers and filter out the key drivers. Raw data and related information can be obtained through experts who have reasonable knowledge and are aware of lean construction from various fields of the construction industry, e.g., supervisors, managers, contractors, or even academicians, through questionnaire surveys.

To achieve the study objective, first, a questionnaire with a 7-point Likert scale was developed to identify the importance of each driver. The respondents were asked to indicate the level of importance of each driver that can motivate stakeholders, managers, or decision-makers to implement lean construction strategy within construction projects based on the following scales; 1 = Not at all important, 2 = Low importance, 3 = slightly important, 4 = Neutral, 5 = moderately important, 6 = very important, and 7 = extremely important. Then, to isolate the key drivers based on the experts’ point of view, the rate of influence measured using a scale of 1 to 7 asked at the end of the questionnaire. The study conducted in January 2019 in Malaysia engaged twenty-three experts in the field of lean construction. Respondents were interviewed and requested to range from 1 to 7 the level of importance of each driver based on the three distinct aspects of sustainable construction (economic, social, and environmental). Table 2 presents the demographics of the respondents.

**Table 2:** Experts’ profile.

Measure	Item	Frequency	Percentage
**Job Position**	Consultant	3	13
	Contractor	5	22
	Project manager	3	13
	Managing director	2	9
	Head of the technical department	6	26
	Academic	4	17
**Years of experience**	Under 6 years	4	17
	6 to 10 years	3	13
	11 to 15 years	5	22
	16 to 21 years	6	26
	Above 21 years	5	22
**Company Type**	Design	2	9
	Construction	9	39
	Both design and construction	5	22
	Consultant	3	13
	Others	4	17

In order to ensure about quality and reliability of gathered data, the Cronbach’s alpha was employed to evaluate this importance. In this regard, Cronbach’s alpha test was performed for each group of drivers. The results showed that the total Cronbach’s alpha value for all groups is greater than 0.7, which proves the reliability of obtained data from the questionnaire [142].

**Fig 3:**
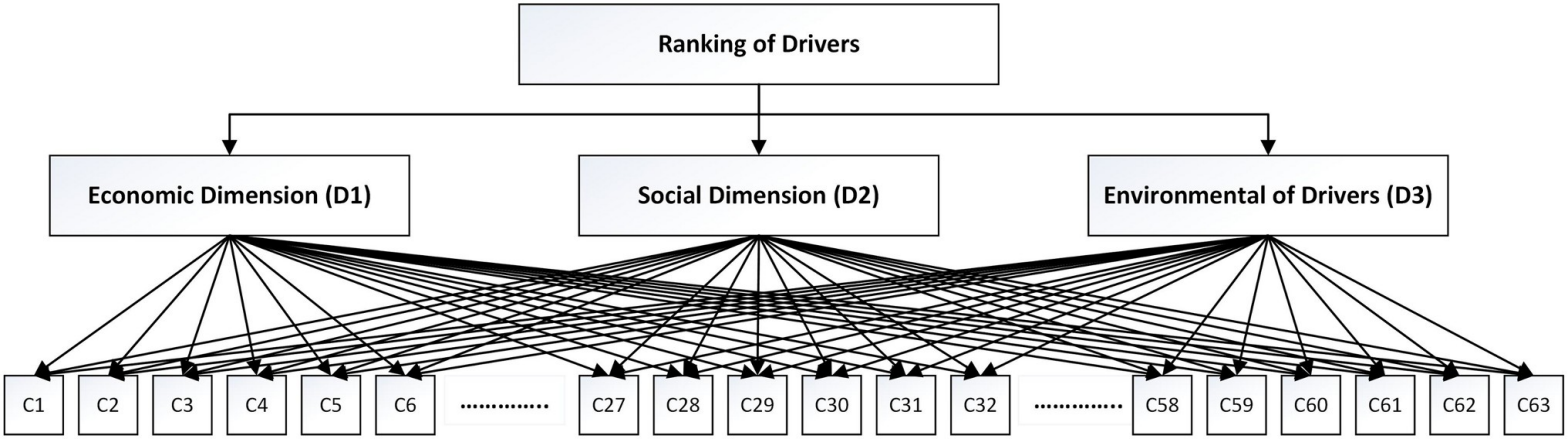
A hierarchical structure of three criteria to rank 63 presented drivers.

The results of this section were further analyzed with weighted TOPSIS and entropy techniques. The TOPSIS technique was applied to rank all drivers based on the closeness to the ideal solution, while the entropy method was engaged in calculating the weight of each indicator (economic, social, and environmental). Fig 3 shows the hierarchical structure of three criteria to rank 63 presented drivers. After ranking all drivers, the values higher than the obtained threshold from the questionnaire were filtered out and then introduced as key drivers.

### Entropy and TOPSIS analysis to prioritize the drivers

In this study, TOPSIS was engaged to rank the significant drivers based on their influence in the implementation of lean construction. Also, this statistical analysis was used to establish the robustness and performance of the proposed methods in this study. Based on the 63 drivers listed in Table 1 and Fig 2, the first questionnaire was designed to gather data from 23 experts, who were active in construction projects. The respondents were asked to rate the importance of each driver in the implementation of sustainable lean construction using a 7-point Likert scale. Upon completing the data collection stage, an initial decision matrix constructed for each expert respondent:

$$A={(x}_{ij}{)}_{63\times 3}=\left[\begin{array}{ccc} 7 & \cdots & 6\\ \vdots & \ddots & \vdots \\ 5 & \cdots & 3 \end{array}\right]$$

To obtain an average rating of expert’s opinions, the matrix of $z={\left[a_{ij}\right]}_{63\times 3}$ was calculated by employing Eq 1

$$z={(a}_{ij}{)}_{63\times 3}=\left[\begin{array}{ccc} 5.8 & \cdots & 5.6\\ \vdots & \ddots & \vdots \\ 5.9 & \cdots & 6.3 \end{array}\right]$$

To acquire a standardized decision matrix, all values were normalized by Eq (2):

$$R=(r_{ij}{)}_{63\times 3}=\left[\begin{array}{ccc} 0.005 & \cdots & 0.008\\ \vdots & \ddots & \vdots \\ 0.005 & \cdots & 0.008 \end{array}\right]$$

To calculate the weight of the indicators (economic, social, and environmental), which was needed for Eq (3), the entropy method used. Therefore, the weight of each indicator was calculated using Eqs (9)–(12). The result of this process helped to develop the matrix.

$$w_{1}=0.18,w_{2}=0.31,w_{3}=0.51\to w_{j=}{\left[\begin{array}{ccc} 0.18 & 0 & 0\\ 0 & 0.31 & 0\\ 0 & 0 & 0.51 \end{array}\right]}_{3\times 3}$$

Where, *w*_1_, *w*_2_, and *w*_3_ are the weight of economic (D1), social (D2), and environmental (D3) indicators, respectively.

Subsequently, using Eq (3) to calculate the weighted normalized decision matrix resulted in:

$$V=\left[\begin{array}{ccc} w_{1}\;r_{11} & \cdots & w_{n}\;r_{1n}\\ \vdots & \ddots & \vdots \\ w_{1}\;r_{m1} & \cdots & w_{n}\;r_{mn} \end{array}\right]={\left[\begin{array}{ccc} 0.0010 & \cdots & 0.0033\\ \vdots & \ddots & \vdots \\ 0.0010 & \cdots & 0.0037 \end{array}\right]}_{63\times 3}$$

Next, the ideal solution calculated by Eqs (4) and (5).

*A*^+^ = 0.0011, 0.0022, and 0.0038

*A*^−^ = 0.0008, 0.0014, and 0.0021

Finally, the closeness coefficients were calculated using Eqs (6)–(8). The drivers were then ranked as illustrated in Table 3.

**Table 3:** Final ranking of the 63 identified drivers.

Rank	Driver’s Code	Dsi-[Dsi++Dsi-]	Rank	Driver’s Code	Dsi-[Dsi++Dsi-]
25	C1	0.6561	4	C33	0.8607
2	C2	0.8854	52	C34	0.3782
62	C3	0.1647	49	C35	0.3867
34	C4	0.5553	8	C36	0.8417
57	C5	0.2909	13	C37	0.8116
63	C6	0.1165	46	C38	0.4650
60	C7	0.2577	44	C39	0.4890
61	C8	0.2384	55	C40	0.3333
17	C9	0.7677	45	C41	0.4885
51	C10	0.3788	53	C42	0.3649
15	C11	0.7906	22	C43	0.7143
58	C12	0.2889	6	C44	0.8475
33	C13	0.5661	29	C45	0.5919
59	C14	0.2798	43	C46	0.4937
48	C15	0.3969	38	C47	0.5307
20	C16	0.7498	42	C48	0.4988
11	C17	0.8305	40	C49	0.5202
5	C18	0.8598	18	C50	0.7609
56	C19	0.3169	3	C51	0.8630
35	C20	0.5474	21	C52	0.7285
50	C21	0.3797	47	C53	0.4375
19	C22	0.7525	24	C54	0.6711
14	C23	0.7914	30	C55	0.5851
31	C24	0.5826	9	C56	0.8383
26	C25	0.6363	36	C57	0.5440
37	C26	0.5416	27	C58	0.6282
10	C27	0.8308	23	C59	0.7062
12	C28	0.8287	1	C60	0.9031
39	C29	0.5244	16	C61	0.7684
54	C30	0.3629	28	C62	0.6197
41	C31	0.5142	7	C63	0.8470
32	C32	0.5791			

**Fig 4:**
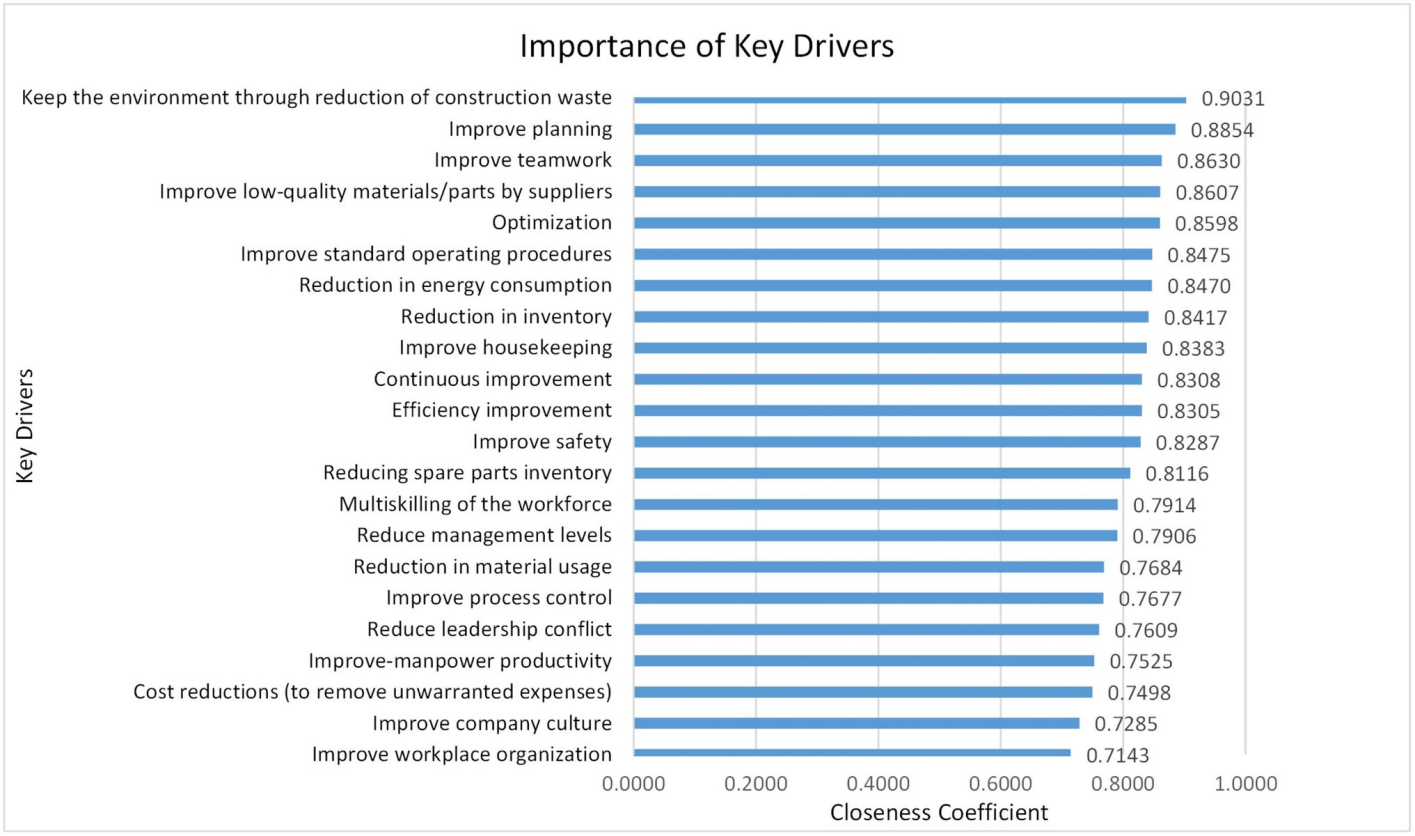
Ranking of key drivers for successful lean construction implementation.

Next, to identify the key drivers, drivers greater than the threshold were filtered out. As previously explained, to attain a threshold based on the experts’ point of view, the rate of influence, which was measured based on a scale of 1 to 7, asked at the end of the questionnaire. In this study, based on the experts’ viewpoint, a rate of 0.71 was set to identify the key drivers. As such, values greater than the threshold value of 0.71 (as shown in Table 3) are extracted and depicted in Fig 4.

**Fig 5:**
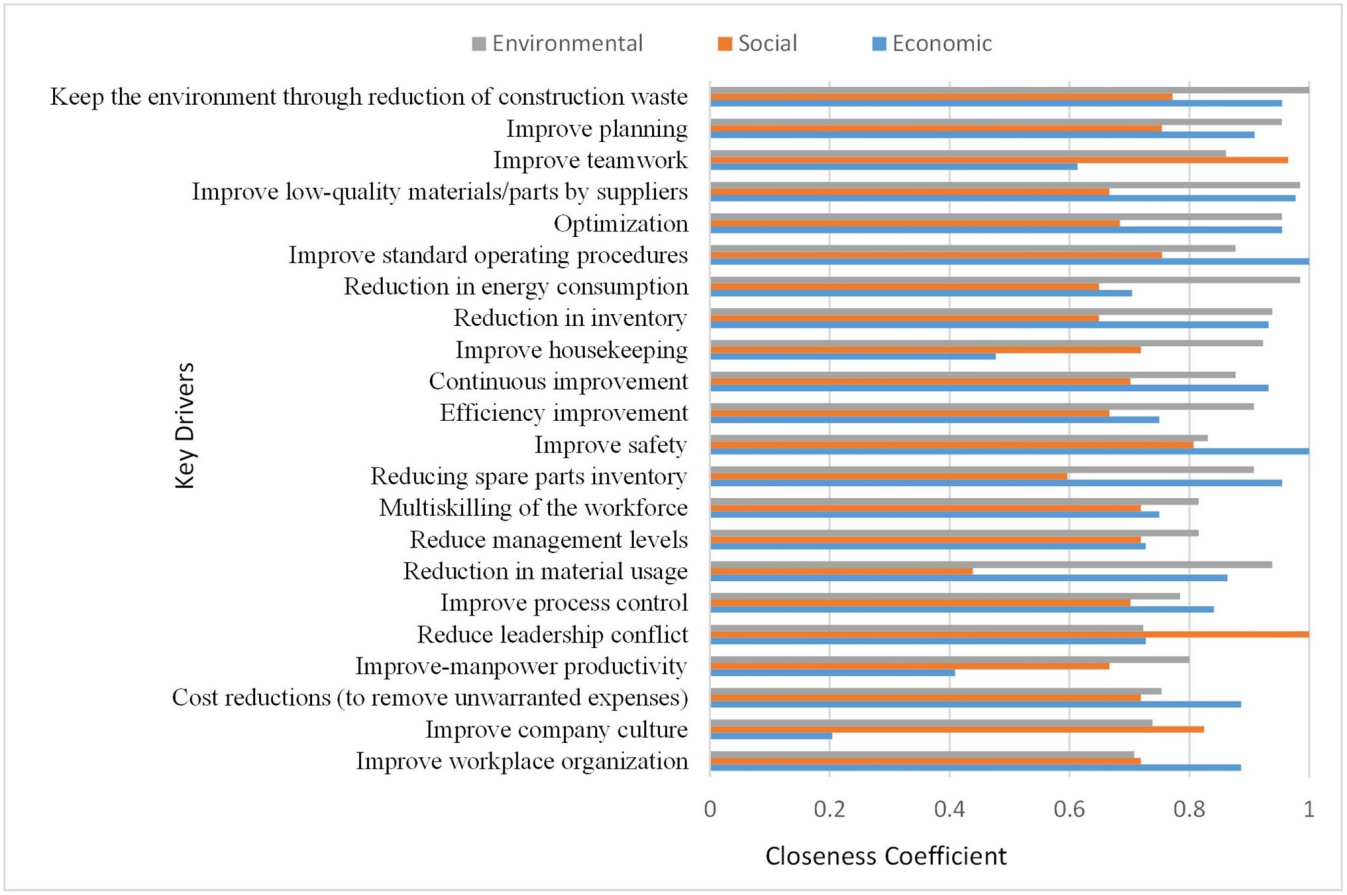
Closeness coefficient for key drivers according to individual perspective.

Fig 4 shows the relative importance index of all key drivers for the successful implementation of lean construction within the Malaysian construction industry, in which 22 key drivers categorized into different classifications. Likewise, the closeness coefficients for each key driver based on individual perspective is also calculated and presented in Fig 5. In other words, Fig 5 shows a cumulative ranking of the drivers based on a single dimension when the weight of each dimension is equal to 1. Therefore, Fig 5 can give an insight to decision-makers to only base on the ranking of each driver in a specific dimension without considering the weight of indicators in selecting their key drivers.

## Discussion

The objective of this study was to propose a Multi-Criteria Decision Making (MCDM) approach using the entropy weighted TOPSIS method to rank all important drivers and identify key drivers for a successful and sustainable lean construction implementation. Ultimately, the obtained results would help managers and decisionmakers to implement sustainable lean construction successfully. This section presents the interpretation of the results obtained by the entropy weighted TOPSIS method and a brief discussion on the findings.

Based on an extensive literature review, 63 important drivers related to the implementation of lean construction identified and classified into seven main groups, which were verified by experts. Because of the important role of lean construction in improving sustainability, the attempt was to find all drivers in three aspects of sustainability (social, economic, and environment). Fig 2 shows the final hierarchy structure model of sustainable lean construction implementation drivers. According to the entropy weighted TOPSIS results (as shown in Table 3), the drivers were ranked based on their importance in motivating stockholders, managers, decision-makers, or those active in the construction industry and their effect on each aspect of sustainability (economic, social and environmental) to implement a successful and sustainable lean construction within construction projects. A threshold was then established based on the experts’ viewpoints to identify the key drivers of lean construction implementation. In this study, values (from Table 3) higher than the set threshold were selected and presented in Fig 4. The results showed that based on the experts’ viewpoints, 22 out of the 63 drivers, which selected from different classification groups, plays a vital role in the successful implementation of lean construction strategy in the Malaysian construction industry.

Moreover, the results of closeness coefficients of key drivers according to individual perspective were calculated and presented in Fig 5. The results of Fig 5 depict that the final ranking of drivers and selecting key drivers was based on an aggregate of the effect of each driver on three indicators (economic, social, and environmental). For example, “reduce leadership conflict” has the highest closeness coefficient index in the social dimension. However, this index in the economic and environmental dimensions is lower. The integration of these three coolness coefficients caused the “reduce leadership conflict” placed as eighteenth key drivers. Therefore, it can be concluded all these key drivers have been selected based on their highest positive effect on each aspect of sustainability, which ultimately helps to a successful and sustainable lean construction implementation.

To discuss key drivers, it is appropriate to base the discussion according to their groups and classifications as those classified in the same group are very similar. Due to the insufficient criteria to rank the classification groups based on their importance, they ranked according to their percentage of nominated key drivers. For example, in Fig 4, the environmental group can be considered as the most important driver group based on the percentage of its drivers that nominated as key drivers (60%). The environmental driver group consisted of three key drivers, which were “keep the environment through the reduction of construction waste,” “reduction in energy consumption,” and “Reduction in material usage” and were the first, seventh, and sixteenth most important key drivers in Fig 4, respectively. This shows that the experts believed that construction companies in Malaysia are interested in those methods, which can improve the environmental dimension of sustainability. It might be in conjunction with the recommendation of the Construction Industry Master Plan (CIMP) of Malaysia, which is to use modern methods to help address sustainability [60].

The second most important driver group is the resource group (50% of its drivers selected as key drivers). “improve low-quality material/parts by suppliers”, “reduction in inventory”, and “reducing spare parts inventory” were the fourth, eighth, and thirteenth most important key drivers respectively Their selection might be due to the challenges that Malaysian companies are facing such as inappropriate inventory process [143, 144] and quality failures in the Malaysian construction industry [145, 146].

Awareness and education, as well as people and culture groups placed as third important driver groups, both are having 36% nomination of key drivers. “Continuous improvement,” “improve safety,” “multiskilling of the workforce,” and “improve-manpower productivity” were the tenth, twelfth, fourteenth, and nineteenth most important key drivers respectively. The previous studies have pointed out that “continuous improvement” can be a useful tool for the application of sustainability within the Malaysian construction industry [147, 148] same with the experts in this study who believed continue improvement can help construction contractors in Malaysia to improve their products, services, and processes. The selection of improving safety as a key driver may be due to a majority of Malaysian contractors have a problem in instilling a safety culture among their workers and staff [149, 150]. Inadequate skills and experiences of the workforce being one of the leading causes of delay within the Malaysian construction industry [148, 151], which experts in Malaysia believe that lean construction can encounter this problem through multiskilling of the workforces and improve human resources productivity. On another hand, “improve teamwork,” “Improve housekeeping,” “Reduce leadership conflict,” and “improve company culture” are third, ninth, eighteenth, and twenty-first import key drivers, respectively. Sine one of the significant problems within the Malaysian construction industry is the lack of teamwork among employees [148] and leadership conflict [26]; improving these issues can be sufficient motivation for managers to implement lean construction.

Additionally, Experts believed that enhancing the housekeeping within the Malaysian construction industry can effectively improve safety. Moreover, as mentioned earlier, the lack of company culture has been one of the contributing factors that have failed successful lean construction implementation in various countries [27]. However, lean construction through training, standardizing of the work structure, and increasing organizational commitment can commendably help to improve company cultures such as teamwork and transparency.

The fourth most important driver group for a successful implementation of lean construction is the financial group, with 30%. “Optimization,” “efficiency improvement, “and “cost reduction” ranked in fifth, eleventh, and twentieth among key drivers. Based on the experts’ viewpoint in Malaysia, these drivers can be significant for lean construction implementation since several companies in Malaysia encounter delay [151], low efficiency [152], and cost overrun [153].

The fifth most important driver group is management with a nomination of 27% drivers. “improve planning”, and “Improve process control”, which were the second, and seventeenth most important key drivers can play an essential role in promoting performance [154] and applying sustainability [155] within the Malaysian construction industry. Also, experts in the Malaysian construction industry believed that “Reduce management levels,” which is the fifteenth important key driver, can improve information sharing, reduce leadership conflict, and improve the company culture.

Finally, the technical group is the least important driver group, with only 18% nomination. “Improve the standard of operating procedures” and “Improve workplace organization” were the sixth and twenty-two most important key drivers. Experts believed that these two drivers could have a key role in applying discipline, which helps to improve quality as well as decrease both the cost and duration of construction projects. Although standardization is daunting within the construction industry in Malaysia [156], implementing lean construction can be an effective solution to this problem.

**Fig 6:**
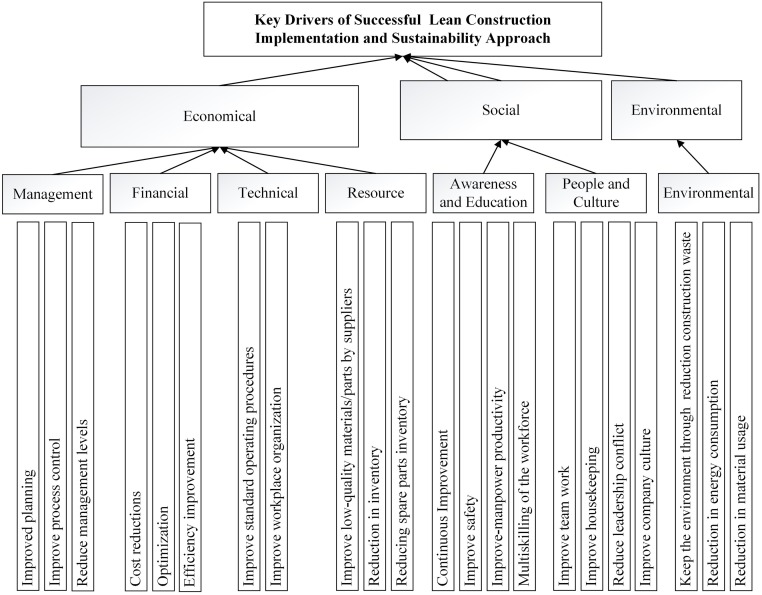
MCDM model of key drivers for successful and sustainable lean construction implementation.

Fig 6 shows an MCDM model using the entropy weighted TOPSIS method, which is constructed based on the results of this study. The model has included the effect of the identified key drivers based on the entropy weighted TOPSIS on successful and sustainable lean construction implementation. The results of this study indicated that the entropy weighted TOPSIS technique can present two useful kinds of information to the decision-makers and managers; (1) ranking of the important drivers and (2) key drivers of lean construction implementation. Through the ranking of drivers and by obtaining a threshold based on the experts’ viewpoint, the key drivers for successful and sustainable lean construction identified. The recommended entropy weighted TOPSIS method can aid in boosting the procedure of key drivers’ identification and promote the success of a sustainable lean construction implementation simultaneously. The entropy weighted TOPSIS also helps to prioritize drivers that should be concentrated on so that the managers and decision-makers can focus on the said key drivers. The proposed methodology in this study is an effective process to assess and evaluate drivers and identify the key drivers for the implementation of lean construction within construction projects with a sustainability approach. The presented tools in this study were based on fundamental theories, which promoted the process of identifying key drivers that can have an essential role in adapting and successfully implementing the lean method in construction projects. The constructed model, which was developed by the two said techniques, can aid in identifying the significant drivers that directly affect the outcomes of a successful and sustainable lean construction implementation.

## Validation of model and data

It is essential to test the validity of data collected and the model developed for its appropriateness. Data and models can be validated through various techniques and tools. The developed model can be validated through qualitative and quantitative methods. Qualitative validation, like comparing the developed model with the results obtained by different techniques the same as Vlse Kriterijumska Optimizacija Kompromisno Resenje (VIKOR) and quantitative validation can be carried out through sensitivity analysis, collection of data with personal interviews, structured questionnaire and sample surveys. However, the validation of data is mostly based on quantitative analysis, like Cronbach’s alpha.

In this research, the key performance measures have been used to establish the validity and utility of obtained data and the proposed model. In this regard, all the identified drivers were first verified by experts through a semi-structured format of Delphi interview. Additionally, the Cronbach’s alpha test was used to validate the reliability and quality of obtained data through an interview by experts. To continue, the identified key drivers were validated through the comparison the outcomes with the VIKOR technique which ultimately lead to validate the proposed MCDM techniques and model in this study. Finally, a sensitivity analysis was conducted to evaluate the influence of criteria weights on the ranking of drivers.

### Comparison with the existing method

To show the validity and feasibility of the suggested method, the VIKOR technique was engaged to compare their results. The reasons for selecting VIKOR technique for comparing the obtained results are: 1) Similar to TOPSIS technique, VIKOR is an MCDM tool for ranking the alternatives based on an aggregating function representing closeness to the reference point (s) [157]. There are several studies in the field of construction management which have used VIKOR technique for factors ranking and selecting an ideal solution. For instance, Ebrahimnejad et al. [158] employed the VIKOR method to rank the high risks in large-scale projects. In another study by Wang et al. [159], the important risks within the construction project were ranked and evaluated using VIKOR approach. Ramezaniyan et al. [160], engaged a fuzzy integration VIKOR-AHP to rank the contractors in a construction industry project. 2) The previous studies have compared the outcomes of TOPSIS method with VIKOR technique to validate the stability and accuracy of the obtained final results [6, 161].

To compare the results of the two mentioned methods, the similar weights of criteria were used in the computation process of the VIKOR method. The purpose of comparing the two methods was to check whether the same key drivers will be selected even by using another ranking method. However, it is predictable that the ranking of selected drivers may be different because VIKOR method tends to set a compromise solution that provides a maximum group utility for the majority and minimum for opponents and a minimum of an individual regret for the opponent. Whereas, the TOPSIS method is selecting a solution based on the shortest distance from the positive-ideal solution and the farthest distance from the negative ideal solution. Moreover, TOPSIS uses vector normalization while VIKOR applies linear normalization in removing the units of criterion functions. Table 4 present the ranking of 22 key drivers based on VIKOR analysis by varying V (V is a weight for the strategy of ‘‘the maximum group utility” or ‘‘the majority of criteria” which is applicable in VIKOR formula for ranking the factors).

**Table 4:** Comparison of TOPSIS ranking with VIKOR ranking for variety of V.

**Key Drivers**		c60	c2	c51	c33	c18	c44	c63	c36	c56	c27	c17	c28	c37	c23	c11	c61	c9	c50	c22	c16	c52	c43
**TOPSIS rank Rrranking**		1	2	3	4	5	6	7	8	9	10	11	12	13	14	15	16	17	18	19	20	21	22
**VIKOR rankings for variety of V**	0.1	1	3	2	11	7	4	14	13	8	6	12	5	17	9	10	22	15	19	16	18	20	21
	0.2	1	2	3	8	7	4	14	12	9	6	13	5	16	10	11	22	15	19	17	18	21	20
	0.3	1	2	3	7	6	4	12	9	10	8	13	5	16	11	14	22	15	19	17	18	21	20
	0.4	1	2	3	7	6	4	10	9	12	8	11	5	15	13	14	21	16	17	19	18	22	20
	0.5	1	2	4	5	7	3	10	9	12	8	11	6	15	13	14	21	16	17	19	18	22	20
	0.6	1	2	4	5	6	3	10	9	13	8	11	7	12	14	15	20	17	16	21	18	22	19
	0.7	1	2	6	3	5	4	10	8	13	9	11	7	12	14	16	19	17	15	21	18	22	20
	0.8	1	2	6	3	5	4	10	8	14	9	12	7	11	15	16	19	17	13	21	18	22	20
	0.9	1	2	7	3	4	5	10	8	14	9	12	6	11	15	16	19	17	13	21	18	22	20
	1	1	3	7	2	4	5	10	8	14	9	13	6	11	15	17	16	18	12	21	19	22	20

Table 4 shows that in the ten variety of V, still, all the identified key drivers through TOPSIS are among 22 top drivers in VIKOR ranking. Therefore, in brief, the proposed entropy weighted TOPSIS is an effective and reasonable method to rank the presented drivers in this study based on the three criteria of sustainability (economic, social, and environmental) and identify key drivers for a successful and sustainable lean construction implementation. Subsequently, based on the results in Table 4, C60 (Keep the environment through the reduction of construction waste) is the optimal key driver. Also, C2 (improve planning) with 8 times the frequency of occurrence is the second import key driver.

### Sensitivity analysis

To investigate the robustness of the ranking and selection of key drivers for a sustainable lean construction implementation, a sensitivity analysis that includes 9 experiments is performed based on the weight change of each criterion. Sensitivity analysis based on the weight change of indicators is the most common method and a necessary step that is used to verify the feasibility and reliability of a model or a method [162]. Using Sensitivity analysis based on weight change is becoming progressively widespread in many fields of sciences and engineering [163]. The said method is one of the most frequent tools which has been used to verify the stability and accuracy of the attained final results by TOPSIS technique. For example, the study which was conducted by dos Santos et al. [35] on performance evaluation of green suppliers using entropy-based TOPSIS, the sensitivity analysis was used to investigate impact of the weight of the indicators for the selection of suppliers. Wood [164] applied sensitivity analysis based on the weight change for Supplier selection for development of petroleum industry facilities using entropy weighted TOPSIS. Li et al. [162], through the theoretical analysis and case study, proved that the TOPSIS method in water quality evaluation is a reliable and feasible technique concerning the sensitivity analysis to weights. Therefore it can conclude that sensitivity analysis based on the weight change of criteria is a reliable tool to check the accuracy and consistency of obtained results. In fact, the sensitivity analysis investigates the impact of each criterion weight change on the ranking of drivers. For assuming, if the weight of the Nth criteria changes from *w*_*n*_ to $w_{n}^{\prime }$ as:

$$w_{n}^{\prime }=w_{n}+\Delta_{n}$$

Then, the weight of other criteria would change to $w_{j}^{\prime }$ whereas:

$$w_{j}^{\prime }=w_{j}+\Delta_{j};j=1,2,‥,k,j\neq n$$

And the new weight of other criteria can be obtained by the following formula:

$$w_{j}^{\prime }=\frac{1-w_{n}^{\prime }}{1-w_{n}}.w_{j}$$ (13)

Table 5 shows for each criterion three conducted experiments (0.2, 03, and 0.4). For each change in the weight of criteria, the weight changes of other criteria have been calculated based on the Eq (13). For example in the third experiment, after the increase of the weight of first criteria by Δ_3_ = 0.4 the weight of other criteria decreased to 0.16 and 0.26. For each condition, closeness indices of each driver and the changes in the final ranks of 63 drivers are calculated and presented in Table 5.

**Table 5:** The 9 experiments of sensitivity analysis.

Expt. No.	Description	New Weight			Ranking
		$D_{1}^{\prime }$	$D_{2}^{\prime }$	$D_{3}^{\prime }$	
1	$D_{1}^{\prime }{}_{=}\;D_{1}+0.2$	0.38	0.23	0.39	C60>C2>C33>C18>C44>C36>C28>C27>C37>C63>C17>C51> C23>C61>C11>C9>C16>C56>C50>C43>C22>C1>C25>C52>C24>C59>C45>C32>C62>C54>C13>C20>C47>C4>C26>C46>C55>C39>C58>C57>C41>C31>C38>C49>C29>C15>C21>C35>C10> C48>C42>C53>C19>C34>C40>C5>C30>C12>C3>C14>C8>C7>C6
2	$D_{1}^{\prime }{}_{=}\;D_{1}+0.3$	0.48	0.20	0.32	C60>C2>C33>C18>C44>C28>C36>C27>C37>C17>C63>C9>C61>C16>C23>C11>C43>C51>C50>C56>C1>C24>C25>C45>C32>C22>C13>C47>C20>C46>C39>C62>C4>C26>C41>C52>C15> C54>C21>C38>C59>C57>C35>C55>C31>C49>C10>C19>C42> C58>C29>C34>C3>C53>C48>C5>C40>C12>C8>C14>C7>C30> C6
3	$D_{1}^{\prime }{}_{=}\;D_{1}+0.4$	0.58	0.16	0.26	C60>C33>C44>C18>C2>C28>36>C27>C37>C16>C61>C9>C43>C17>C23>C63>C11>C50>C51>C24>C25>C47>C1>C32>C45>C46>C13>C39>C20>C56>C15>C21>C41>C26>C4>C35>C38> C22>C19>C62>C10>C42>C57>C3>C31>C54>C52>C55>C49> C34>C59>C5>C29>C8>C58>C12>C40>C14>C53>C7>C48>C30>C6
4	$D_{2}^{\prime }{}_{=}\;D_{2}+0.2$	0.13	0.51	0.36	C51>C60>C50>C28>C2>C44>C56>C18>C52>C27>C33>C23> C11>C54>C63>C17>C36>C9>C16>C43>C22>C37>C57>C58> C49>C55>C61>C48>C62>C32>C31>C25>C24>C53>C59>C45> C1>C29>C13>C20>C41>C47>C30>C26>C4>C42>C39>C15> C38>C10>C46>C21>C19>C35>C7>C34>C8>C12>C40>C5>C14>C6>C3
5	$D_{2}^{\prime }{}_{=}\;D_{2}+0.3$	0.10	0.61	0.29	C51>C50>C28>C60>C54>C52>C2>C44>C57>C56>C23>C11> C27>C18>C49>C16>C33>C9>C43>C17>C63>C36>C55>C22> C58>C48>C37>C31>C53>C62>C32>C24>C61>C25>C45>C29> C1>C59>C30>C13>C42>C41>C15>C10>C20>C19>C21>C38> C47>C39>C26>C46>C35>C4>C7>C8>C34>C12>C40>C5>C6> C3>C14
6	$D_{2}^{\prime }{}_{=}\;D_{2}+0.4$	0.08	0.71	0.21	C51>C50>C54>C57>C28>C52>C49>C60>C2>C44>C56>C23> C11>C55>C16>C48>C43>C27>C9>C18>C58>C33>C17>C22> C63>C36>C53>C31>C37>C62>C32>C24>C25>C29>C45>C30> C61>C42>C19>C15>C10>C41>C1>C13>C21>C20>C59>C38> C39>C35>C47>C46>C7>C26>C8>C4>C12>C6>C34>C5>C40> C3>C14
7	$D_{3}^{\prime }{}_{=}\;D_{3}+0.2$	0.11	0.18	0.71	C60>C2>C33>C63>C18>C36>C56>C17>C37>C61>C44>C27> C51>C59>C28>C23>C11>C22>C9>C16>C52>C50>C1>C43> C25>C54>C58>C62>C4>C45>C13>C26>C24>C20>C32>C55> C47>C29>C46>C39>C41>C31>C38>C57>C48>C49>C34>C53> C35>C15>C40>C21>C10>C30>C42>C14>C5>C12>C19>C7> C8>C3>C6
8	$D_{3}^{\prime }{}_{=}\;D_{3}+0.03$	0.07	0.12	0.81	C60>C33>C63>C2>C18>C36>C56>C61>C17>C37>C59>C44> C27>C51>C28>C23>C11>C22>C9>C16>C52>C1>C50>C43>C25>C54>C58>C62>C4>C45>C13>C26>C24>C20>C32>C47>C55>C29>C46>C39>C41>C31>C38>C57>C48>C49>C34>C53>C35> C40>C15>C21>C10>C30>C14>C42>C5>C12>C19>C7>C8>C3>C6
9	$D_{3}^{\prime }{}_{=}\;D_{3}+0.4$	0.03	0.06	0.91	C60>C33>C63>C2>C18>C36>C61>C56>C59>C17>C37>C44> C27>C51>C28>C23>C11>C22>C9>C16>C52>C1>C50>C43>C25>C54>C58>C62>C4>C45>C13>C26>C24>C20>C32>C47>C55>C29>C46>C39>C41>C31>C38>C57>C48>C49>C34>C53>C35> C40>C15>C10>C21>C30>C14>C42>C5>C12>C19>C7>C8>C3>C6

According to the results presented in Table 5, the following conclusions can be obtained: 1) out of nine experiments, driver C50 (Keep the environment through reduction of construction waste) has scored highest in six experiments; hence, Keep the environment through reduction of construction waste is recommended as the most significant driver for the implementation a sustainable lean construction implementation; 2) the ranking of 63 drivers is relatively sensitive to the criteria weights; 3) the final ranking of the drivers vary significantly with the weight changes of each criterion; and 4) the proposed methodology can effectively robust the selection of key drivers for a successful and sustainable lean construction implementation.

## Conclusion

On the whole, lean construction is a feasible and strategic approach, which not only promotes productivity but also helps to obtain sustainability during construction projects because it includes a clear set of objectives for the process of delivery. However, previous studies have shown that the process of adaption and implementation was poor at low speed or showed no progress. Drivers of lean construction implementation can motivate managers to adapt and successfully implement lean construction effectively. However, previous studies in the field of lean construction have not adequately focused and attempted to presenting all important drivers or proposing a methodology for identifying the key drivers of a successful and sustainable lean construction implementation. Hence, this study suggested a novel methodology by employing entropy weighted TOPSIS technique to identify and classify important drivers based on the three criteria of sustainability (economic, social, and environmental) and ascertain key drivers for successful and sustainable lean construction implementation. It showed that entropy weighted TOPSIS was a useful approach to rank the significant drivers to identify key drivers based on closeness index, which in this regard entropy was used to calculate the weight of the indicators and therefore overcome the shortage of subjective weighting and prevent the effect of human subjective factors. The results of this study showed the following:

1. A large number of 63 drivers of lean construction implementation in three dimensions of sustainability (social, environmental, and economic) were identified and verified by seven Malaysian construction professionals.
2. A hierarchical structure of drivers for a sustainable lean construction implementation proposed which all drivers classified into seven main groups (management, financial, technical, resource, awareness and education, environmental, people, and culture).
3. All the drivers were ranked based on their closeness to the positive ideal solution, and the outcome showed that the experts in the Malaysian construction industry believed that 22 out of the 63 drivers have a key role in successful and sustainable lean construction implementation.
4. All driver groups were ranked based on the percentage of nominated drivers as key drivers, in which in this regard, the environmental driver group identified as the most significant group.
5. An MCDM model of key drivers for successful and sustainable lean construction implementation proposed.
6. The proposed method and model in this study were validated through a comparison between similar techniques for ranking (VIKOR) and also by employing sensitivity analysis.
7. The proposed novel methodology with employing entropy weighted TOPSIS was a reasonable and practical approach for identifying key drivers of lean construction implementation, which can be applied in other studies for finding the key factors (e.g., key success factors, key barriers, etc.).

This study can be useful for researchers in the field of lean construction implementation due to its ability to present a checklist of essential drivers and a framework of key drivers for the successful implementation of lean construction with sustainability. Furthermore, the results of this study can help managers and policymakers in Malaysia by boosting the process of decision making in identifying key and critical drivers for successful and sustainable lean construction implementation. Also, these outcomes aid decision-makers to concentrate on the most significant drivers of lean construction implementation within the construction industry. Moreover, this paper not only presents the key drivers for the successful implementation of lean construction in Malaysia but also highlights the problems with which construction industries in Malaysia are faced. Understanding these problems helps the government and decision-makers to select the best possible strategy for promoting the efficiency and productivity of Malaysian construction industry. It is anticipated that these findings can contribute to the successful implementation of lean construction and would promote awareness and bridge the gap between theory and practice to achieve sustainable lean construction successfully.

Future studies can evaluate and analyze the interaction and interrelationships between the identified key drivers in this study using MCDMS techniques such as Decision Making Trial and Evaluation Laboratory (DEMATEL) or Interpretive Structure Modeling (ISM). Developing a framework considering the complex relationships between key drivers can be a useful future extension.

## Supporting information

S1 File Definition of each lean driver. (DOCX)

S2 File Delphi interview results. (DOCX)
