# Supplementary material for: A hybrid approach using entropy and TOPSIS to select key drivers for a successful and sustainable lean construction implementation
Source: PLoS One. 2020 Feb 5;15(2):e0228746. doi: 10.1371/journal.pone.0228746 (PMC7001944; doi:10.1371/journal.pone.0228746)
Supplement: S1 File — (DOCX) [file pone.0228746.s001.docx]

| NO | | **Drivers** | **Definition** |
| --- | --- | --- | --- |
| 1 | | Improve scheduling | Scheduling promotes a decision that define when and who will do the job or activity. Lean through principles (like Flow, value stream and perfection) and techniques (like Last Planner System) can improve the scheduling. |
| 2 | | Improve planning | Planning is a decision for what, how and time estimate for a job. Planning of a job should be done before Scheduling a job or activity. Lean through principles (like Flow, value stream and perfection) and techniques (like Last Planner System) can improve the Planning. |
| 3 | | Global competition | Lean construction through improve standards, quality, decrease in total cost, and shorten the project duration, helps the companies to be active in international markets. |
| 4 | | More focused on organization structure | Lean through the standardization, Improve the typically hierarchical arrangement of lines of authority, communications, rights and duties of an organization. |
| 5 | | Promote prompt and reliable delivery to customer | Reliable delivery is the ratio of the number of deliveries made without any error (regarding time, place, price, quantity, and/or quality) to the total number of deliveries in a period. Lean can reach to this objective through improve standardization and reduce the waste via different tools such as JIT,LPS, First Run studies, etc. |
| 6 | | Short time to fulfill customer orders | Implementing Value stream, Flow and Pull principles lead to company making the process flow, reducing inventories, and reducing waiting time of products between the single steps of the production line |
| 7 | | Promote ability in frequent changes in order by customers | Implementing Pull and Perfection principles can make a company to be flexible regarding the demand of the customer. |
| 8 | | Meeting customer expectation | The principle of Perfection pursues to deliver exactly what the customer desire with regards to quantity and quality, at the right time at a cost-effective and with minimum waste, in which the real goal is zero waste. |
| 9 | Improve process control | | Last Planner System (LPS) and Fail Safe for Quality and Safety are effective tools of lean construction in improving process control within construction projects. |
| 10 | Improve the production capacity of the company | | Lean construction through principles and techniques help project to reach the maximum level of output possible. Lean construction increases the production capacity by increasing efficiency and productivity. Improvements in efficiency can often reduce utilization as more output is achieved with fewer resources in less time. |
| 11 | Reduce management levels | | Lean construction attempts that structure of organization to become more flat by reducing or eliminating the hierarchy which lead to a business encouraging better information sharing across the organization. Limiting the hierarchy also closes the traditional gap between management and workers, which reduces the friction that usually accompanies that gap. Rather than fixating on how to secure a promotion, workers spend their energy on maximizing their professional skills. |
| 12 | Increase market share | | Market share represents the percentage of an industry or market's total sales that is earned by a particular company over a specified time period. This advantage can achieve through price competition. Lean help to increase market share through cost reduction. |
| 13 | Increase flexibility | | Lean construction is able to increase flexibility of company through principles and tools wherein company is able to respond to potential internal or external changes affecting its value delivery, in a timely and cost-effective manner. |
| 14 | Reduce high-labor-cost or labor requirements | | lean decrease labor requirement by automation, improve process, standardization, etc. |
| 15 | Cost savings | | Cost Savings are actions and methods that will result in fulfillment of the objectives and at a cost lower than the historical cost. Lean Construction through perfection principle and also thorough the tools such as: First Run Studies, Concurrent Engineering, KAIZEN, and LPS help in cost saving. |
| 16 | Cost reductions | | Cost reduction is the process of looking for, finding and removing unwarranted expenses from a process to increase profits without having a negative impact on product quality. Lean process help to cost reduction through elimination of non-value adding activities. |
| 17 | Efficiency improvement | | Efficiency is the comparison of what is actually produced or performed on what can be achieved with the same consumption of resources (money, time, labor, etc.). Improving efficiency is one of the main objectives of lean process. |
| 18 | Optimization | | Lean can improve optimization through finding an alternative with the most cost effective or highest achievable performance under the given constraints, by maximizing desired factors and minimizing undesired ones. |
| 19 | Improve profit margin | | Profit margin is part of a category of profitability ratios calculated as net income divided by revenue, or net profits divided by sales. Lean is able to improve profit margin through finishing the project on time, removing the wastes, improve efficiency, etc. |
| 20 | Mitigation project risk | | Last Planner System is an effective way that helps in mitigation risks. All possible risk can be identified through pre planning and long term planning and effectively treat risks during the project. |
| 21 | Competitive advantage | | Lean can help the organization to be competitive through providing the same value as its competitors but at a lower price, or charging higher prices by providing greater value through differentiation. Competitive advantage results from matching core competencies to the opportunities |
| 22 | Improve-manpower productivity | | Manpower productivity is the rate of output per worker (or a group of workers) per unit of time as compared to an established standard or expected rate of output that should be improved. Labor productivity can be improved through improve flow, improve planning and standardization. Improve labor productivity leads companies to use less number of manpower. |
| 23 | Multi skilling of the workforce | | Promotes workers who have a range of skills or knowledge for working on several different projects, which may or may not be a part of the worker's technical job description. Lean through the training increase the multi-tasking of workforces which lead to improve flow, labor cost savings, reduction in required workforce, and increase in average employment duration. |
| 24 | Improve capabilities | | Capability is the measure of the ability of an entity (department, organization, persons, and systems) to achieve its objectives, especially in relation to its overall mission. Standardization is one of best way to improve this capability. |
| 25 | Promote skilled workers | | Lean through Training of workers affects them to become knowledgeable about a specific skill or profession. |
| 26 | Commitment to self-action teams | | Lean philosophy attempt removing problems without referring to management through discussion and exchange of views within members of the team in or direction to where the team goes. This as an effective way that help managers to focus more on important problems. |
| 27 | Continuous Improvement | | Continuous improvement is defined as a culture of sustained improvement targeting the elimination of waste in all systems and processes of an organization. Continuous improvement is a core component for implementing perfection principle. |
| 28 | Improve safety | | Lean construction can improve safety through different principles and tools such as Value Stream Mapping, Flow, Perfection, 5S, LPS, Fail Safe for Quality and Safety, etc. |
| 29 | Enhanced organization reputation | | Organization reputation has been defined as the ability of the company to deliver improving business results. Lean construction through perusing value adding, meeting customer expectation, removal of waste, on time delivery, etc. can promote organization reputation. |
| 30 | Facility of understanding the concepts of lean construction | | Understanding the concept will cause a positive effect through time and enhances the management process of the projects. Throughout the concept, the waste elimination and value enhancement in a construction project will be defined. This approach is able to make the process of implementing activities in the project in a systematic, organize and effective manner. |
| 31 | Promote awareness of some or all of tools and techniques | | Well-informed about tools and techniques helps in choosing the right tool, in the right place and in the right time that seeks to accomplish project goals. |
| 32 | | Employee autonomy | Autonomy in management is allowing a great deal of freedom to employees to control their work situation. A manager who allows the employees to have autonomy generally let the employee to decide the best way to achieve the project goal. Lack of employee autonomy lead to staff members feeling dissatisfied. Traditionally, only employees in upper management have much autonomy, but lean process through standardized work procedures, improved organization structure, and reduced or eliminated the hierarchy has helped to improve employee autonomy. |
| 33 | | Improve Low-quality material/parts by suppliers | Lean construction through defining supply planning and controlling, and standardized the work procedures can ensure the quality of materials. |
| 34 | | Improve on time delivery by the supplier | The efficiency of supply chain process is measured by on time delivery. This advantage can be achieved by specification of the resources and supply planning; issuing and transmission of purchase requests; transport and receipt of the goods at the construction site; maintenance of the supply forecast in the planning (control and reprogramming), Improve Value Stream Mapping, using Kanban tools, and close commitment with supplier. |
| 35 | | Improve supply reliability | Confidence in a supplier's ability to deliver an acceptable product at the required time that will satisfy the customer's needs. This can be achieved by defining a supply planning, standardizing the work procedures, commitment with contract on a long-term run with long-term contracts, train supplier, sharing of ideas between supplier and buyer, etc. |
| 36 | | Reduction in inventory | This can be achieved through using Just In Time practice which suggests the delivery of raw materials at the exact time they are necessary, for the work in process or finished goods. This helps companies for better utilization of resources and reduces material inventories along the production systems. |
| 37 | | Reducing spare parts inventory | This advantage can be approached through a combination of some techniques such as Fail Safe for Quality and Safety, Kanban, and JIT. Fail Safe for Quality and Safety as a detective function that prevent defective in the process as early as possible, Kanban to demand-forecast, and JIT to deliver spare part in exact time that is needed. |

| 38 | | Improve coordination between supplier and company | This can be achieved through training of supplier, Using Daily Huddle meeting techniques, supplier support by company; sharing of ideas between supplier and buyer; work in a rational framework with supplier; etc. |
| --- | --- | --- | --- |
| 39 | | Reduce lead times | Improve flow, Improve value stream, Improve planning and scheduling, using 5s techniques and concurrent engineering, etc. leads to decrease lag times. |
| 40 | | Redesign of processes | First Run Studies use in projects to redesign critical assignment, are actually a part of continuous improvement effort. This tool is based on productivity studies and review the methods of work by redesigning and streamlining the different functions involved |
| 41 | | Improve the commitment of employees | Employee Commitment is the psychological attachment and the resulting loyalty of an employee to an organization. Improve commitment of employees, improve of team working and information sharing can be attained within the training. Moreover, improving the structure of organization by lean process, reducing or eliminating the hierarchy, using Daily Huddle Meetings tools and continuous improvement can be effective in improving commitment of employees. |
| 42 | | High-product variety | Lean process through organizing the process, standardizing the work, improving efficiency and productivity can help the companies to be flexible in producing high variety of products for customers. |
| 43 | | Improve workplace organization | To improve workplace organization, 5S is the best tool which is also called organization method. 5S presents how to organize a work space for effectiveness and efficiency by detecting and storing the items used, maintaining the items and area and sustaining the new order. Usually, the decision-making process is concentrated on standardization, which improves understanding between employees of how they should do the work. |
| 4 | Improve standard operating procedures | | Standardization is a core element within lean construction which has been defined as a procedure specific to operation that describes the activities necessary to complete tasks in accordance with industry or construction regulations, or even just company own standards for running business or project. Standardized work is a method of defining efficient work process that is repeatedly followed by workers. Important goal of standardized work: eliminating wasteful motion and peruse continuous improvement. |
| 45 | Reduce steps of project’s life cycle | | Reduce steps of project’s life cycle is an effective way for reduce lead time and also reduce the waste. Lean method through value stream mapping, standardized the work, eliminate non value adding, improving planning, etc. can help to reduce the steps in projects. |
| 46 | A stronger focus on performance | | Lean method attempts to improve performance in all level of construction project. Lean process try to identify the key factors that have effect on project performance. All lean principles and tools have strong effects on performance. |
| 47 | Improve process layouts | | Lean process can improve process layout through value stream mapping principle and 5S tools. Improving layout help to improve flow within project and decrease transportation waste and reduce lead time. |
| 48 | Improve self-criticism | | Self-criticism means action of finding one’s own faults and shortcomings. This lead to clear view of projected problems and the unlimited capacity to learn from errors since only part of the problem was perceived. Lean process attempts through training improve self-criticism among employees and decreased hierarchy of authority within the projects. |
| 49 | improve transparency among team | | Transparency has been defined as the ability of a production process to communicate with people. Lean construction through training and improve flow can help to promote transparency within the projects. In this regard, the tools such as 5S, Kanban, Daily Huddle Meetings and Increased Visualization can help to improve transparency. |
| 50 | Reduce leadership conflict | | Leadership conflicts defined as friction or opposition between leadership resulting from actual or perceived differences or incompatibilities. Lean process can decrease this conflict through training, promoting the standardized work, and improve team working and information sharing. Tools such as Daily Huddle Meetings and LPS are effective in preventing leadership conflicts. |
| 51 | | Improve team working | Team working means a culture that workers or employee work collaboratively with a group of people in order to achieve a goal. Team working is a key component of lean construction. Team working can be achieved through training the employees, improve the structure of organization, standardized the work, and encourage employee to commitment. The tools such as 5S, Daily Huddle Meetings and LPS are effective for improving team working. |
| 52 | | Improve company culture | Culture is a key component in successful implementation of lean method. Company culture can be defined as corporate culture pertaining to the beliefs and behaviors that determine how a company's employees and management interact, handle or overcome with problem encountered. Generally, scholars claimed that implementing lean principles and techniques allow people to experience lean and act as a vehicle for the cultural shift of the whole organization (Bortolotti et al., 2015). . |

| 53 | Improve trust | The meaning of trust is reliance on the integrity, strength, ability, surety, etc., of a person or group of people that lead to improve team working. Implementing lean principles and techniques generally lead to improve trust within the employees and manager. Furthermore, lean training, standardized work, structure of organization, improve self-criticism, and improve communication helps to improve trust. The tools such as Daily Huddle Meetings are effective in communication and finally improve the trust. |
| --- | --- | --- |
| 54 | Improve information sharing | Efficient information sharing is an effective way to increase the knowledge level of the entire organization. Information sharing also help to identify value through customer feedback and forward the information to the product company. Lean method aims to create a transparent organization so that every things can be seen by everyone. This is made possible visualizing, like the visual planning tool which is the most effective way for communication, information exchange and progress management. Furthermore, Value Stream Mapping (VSM) methodology is useful for Flow of information. |
| 55 | Motivates employees and shapes their behavior | Lean method can improve motivation through structural organization, organizational commitment, improve team working, and improve transparency. Furthermore, through training programs and carefully designed communication it should be possible to manage employee motivation for and interest to Lean. In addition the other factors that can help to improve motivation in implementing lean construction are self-criticism and Multiskilling of the workforce. 5s, Kaizen, and Daily Huddle Meetings are effective tools that can improve the motivation of employees. |
| 56 | Improve housekeeping | Lean process through the 5s can cleanup an organization of the work place. The 5s process (sometimes referred to as the Visual Work Place) is about “a place for everything and everything in its place”. |
| 57 | Increase employee morale | Lean method can improve employee morale through training, standardized the work structural organizations, organizational commitment, improve team working, and improve transparency. An intense focus on productivity can often lead to legal, moral and ethical compromise. |
| 58 | Government policy and regulation | Government Policy and Regulation can encourage company to implement lean construction (for example give loan for lean implementation) can help to meet up standard required in construction projects and also achieve sustainability. |
| 59 | Reduction air pollution | Lean process through improvement on the supply chain system, improve value stream, improve planning, leads to decrease life cycle of project and decrease transportation and movement wastes. Decrease in transportation and movement waste means using less petrol and decrease in producing CO2. In this regard the JIT and prefabrication tool are influence tools for reduction in motion and reduce in pollution. |
| 60 | Keep environment through reduction construction waste | The main goal of lean principles and techniques is waste reduction through enhanced planning and controlling, improve design, decrease life cycle of projects, and improve efficiency of process. These practices help to reduce material usage and also reduce the reworks which lead to reduction in construction waste that highly affected environmental pollution. In this case, prefabrication is an effective tool that helps to control construction waste. |
| 61 | Reduction in material usage | Lean construction is a “way to design production systems to minimize waste of materials, time, and effort in order to generate the maximum possible amount of value," (Koskela et al., 2002). Many of material wastes are related to overproduction which the quantity is greater than required or earlier than necessary. In this case, Pull principle and JIT tool are very effective in preventing over production of wastes. LPS, 5S, prefabrication, and Kanban are other effective tools. |
| 62 | Water efficiency | Lean process improves the supply chain system, improves the efficiency of process, decreases the variability, decreases non value adding, improves value stream, improves planning lead to decrease life cycle of project. These practices tend to use less equipment, decrease in process time, waiting time, transportation and movement wastes which cause less energy (fuel and electricity) consumption. Value Stream and Pull principle, JIT, LPS, 5S, prefabrication, and Kanban tools can be very effective. |
| 63 | Reduction in energy consumption | Lean principles and practices have a key role in reduction waste such as water consuming during construction or workplaces. Lean construction through redesign the process, improve design, planning and control and using new methods within the construction try to decrease in material usage such concrete. Using less material means using less water. Pull principle, JIT, LPS, 5S, prefabrication, and Kanban are very effective in this matter. |
