# Supplementary material for: A hybrid approach using entropy and TOPSIS to select key drivers for a successful and sustainable lean construction implementation
Source: PLoS One. 2020 Feb 5;15(2):e0228746. doi: 10.1371/journal.pone.0228746 (PMC7001944; doi:10.1371/journal.pone.0228746)
Supplement: S2 File — (DOCX) [file pone.0228746.s002.docx]

| Delphi Interview Results | | | | | | | | | | | | | | | | | | | | | | |
| --- | --- | --- | --- | --- | --- | --- | --- | --- | --- | --- | --- | --- | --- | --- | --- | --- | --- | --- | --- | --- | --- | --- |
| **Expert ID** | **Years of Experience in Construction** | **Round 1** | | | | | | | **Round 2** | | | | | | | **Round 3** | | | | | | |
|  |  | **Drivers Verification** | | | | | **Classification Verification** | | **Drivers Verification** | | | | | **Classification Verification** | | **Drivers Verification** | | | | | **Classification Verification** | |
|  |  | **1** | **2** | **3** | **4** | **5** | **Yes** | **No** | **1** | **2** | **3** | **4** | **5** | **Yes** | **No** | **1** | **2** | **3** | **4** | **5** | **Yes** | **No** |
| E1 | More than 16 years |  |  | 10 | 8 | 45 | 60 | 3 |  |  | 8 | 12 | 43 | 63 | 0 |  |  |  | 7 | 56 | 63 | 0 |
| E2 | More than 20 years |  | 3 | 8 | 6 | 46 | 51 | 12 |  |  | 10 | 8 | 45 | 63 | 0 |  |  |  | 7 | 56 | 63 | 0 |
| E3 | More than 19 years |  |  | 8 | 5 | 50 | 61 | 2 |  |  | 5 | 12 | 46 | 63 | 0 |  |  |  | 7 | 56 | 63 | 0 |
| E4 | More than 15 years |  | 1 | 9 | 12 | 41 | 50 | 13 |  |  | 12 | 14 | 37 | 63 | 0 |  |  |  | 7 | 56 | 63 | 0 |
| E5 | More than 18 years |  |  | 8 | 6 | 49 | 51 | 12 |  |  | 6 | 15 | 42 | 63 | 0 |  |  |  | 7 | 56 | 63 | 0 |
| E6 | More than 21years |  | 4 | 11 | 10 | 38 | 49 | 14 |  |  | 18 | 13 | 32 | 63 | 0 |  |  |  | 7 | 56 | 63 | 0 |
| E7 | More than 16 years |  |  | 8 | 11 | 44 | 51 | 2 |  |  | 3 | 15 | 45 | 63 | 0 |  |  |  | 7 | 56 | 63 | 0 |
| Note: 1 denotes “ Least important” and 5 denotes “ Absolute important” | | | | | | | | | | | | | | | | | | | | | | |
